# Isotopic Indications of Late Pleistocene and Holocene Paleoenvironmental Changes at Boodie Cave Archaeological Site, Barrow Island, Western Australia

**DOI:** 10.3390/molecules26092582

**Published:** 2021-04-28

**Authors:** Jane Skippington, Tiina Manne, Peter Veth

**Affiliations:** 1 Archaeology, School of Social Science, University of Western Australia, Perth, WA 6009, Australia; peter.veth@uwa.edu.au; 2 School of Social Science, University of Queensland, Brisbane, QLD 4072, Australia; t.manne@uq.edu.au; 3 Australian Centre for Excellence in Biodiversity and Heritage, University of Wollongong, Wollongong, NSW 2522, Australia

**Keywords:** isotopes, enamel, archaeology, paleoenvironments, herbivores, mammals, macropods, Australia

## Abstract

This paper presents the first application of mammal tooth enamel carbonate stable isotope analysis for the purpose of investigating late Pleistocene–early Holocene environmental change in an Australian archaeological context. Stable carbon (δ^13^C) and oxygen (δ^18^O) isotope ratios were analyzed from archaeological and modern spectacled hare wallaby (*Lagorchestes conspicillatus*) and hill kangaroo (*Osphranter robustus*) tooth enamel carbonates from Boodie Cave on Barrow Island in Western Australia. δ^18^O results track the dynamic paleoecological history at Boodie Cave including a clear shift towards increasing aridity preceding the onset of the Last Glacial Maximum and a period of increased humidity in the early to mid-Holocene. Enamel δ^13^C reflects divergent species feeding ecology and may imply a long-term shift toward increasing diversity in vegetation structure. This study contributes new data to the carbonate-isotope record for Australian fauna and demonstrates the significant potential of stable isotope based ecological investigations for tracking paleoenvironment change to inter-strata resolution.

## 1. Introduction

**Figure 1:**
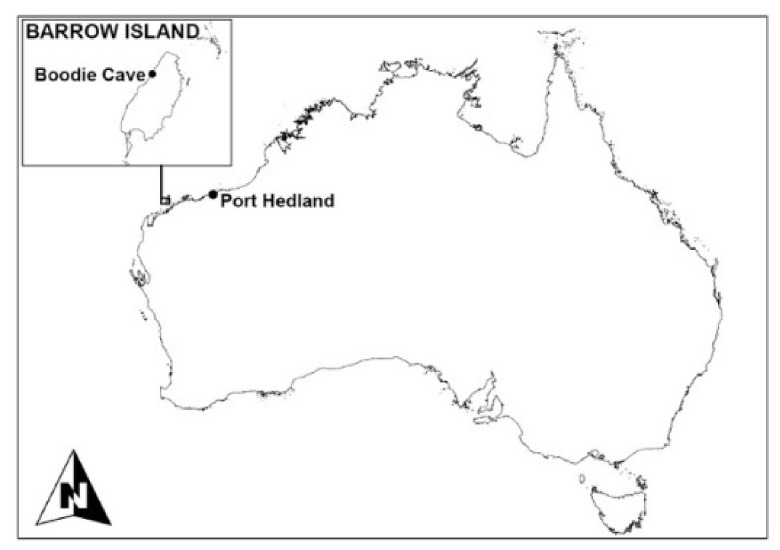
Location of Boodie Cave, Barrow Island, Western Australia.

Boodie Cave is located on Barrow Island in northwest Australia in an area characterized by dynamic precipitation driven by the influence of winter rainfall and tropical cyclones (Figure 1andFigure 2) [1]. Vegetation is dominated by plants utilizing the C4 photosynthetic pathway [2,3]. The archaeological site preserves rich deposits dating from the earliest period of occupation (~50 ky BP) through the post-glacial marine transgression and subsequent islandization (~7 ky BP) [4,5]. This provides a unique opportunity to reconstruct past environments and examine the complex relationships between ecological vectors and human populations during the site’s extensive history.

The notably well-preserved archaeo-fauna assemblages are diverse and contain a varying array of marine and terrestrial resources that suggest a fluctuating mosaic of habitats accompanied broader paleoenvironmental transformations in the area during the late Pleistocene and early Holocene [5,6]. However, the pursuit of further, site-specific paleoenvironmental data is fundamental to expanding understandings of influencing factors including variable precipitation, changing vegetation structure and unstable biological productivity.

Stable isotope geochemistry of archaeological animal tissue represents a well-established, high precision method for extracting local paleoecological data [7,8,9,10,11,12]. The high mineral content and crystalline characteristics of tooth enamel mean that it is highly resistant to diagenetic alteration and thus optimal for isotopic studies of Pleistocene-age archaeological deposits [13,14,15]. 

Utilizing established relationships linking enamel δ^13^C to vegetation structure and δ^18^O to relative humidity in macropods [16,17], this study integrates the isotopic compositions of contemporary and archaeological wallabies (*Lagorchestes conspicillatus*) and kangaroos (*Osphranter robustus*) with stratigraphic analyses to map changing paleoenvironmental conditions at Boodie Cave through time. While there have been a number of isotopic studies undertaken on modern and fossil tooth enamel of Australian macropods [18,19,20,21], this is its first application for creating a high-resolution local isotope record in an archaeological context.

## 2. Materials and Methods

### 2.1. Excavation

**Figure 3:**
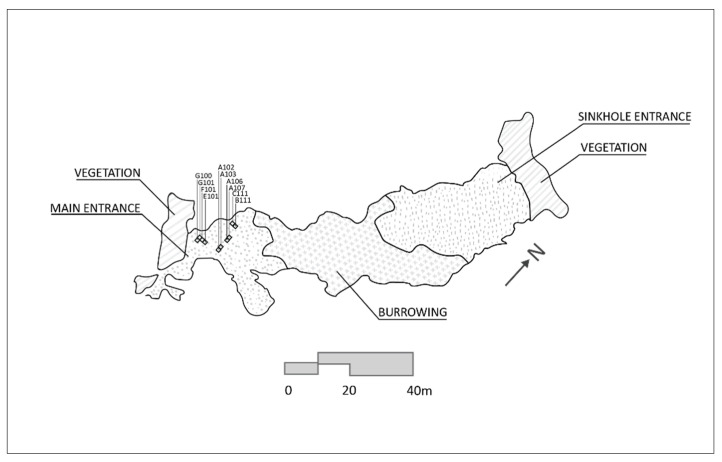
Site plan showing location of squares.

A total of ten 1 m × 1 m squares were excavated at Boodie Cave over the course of three field seasons between 2013 and 2015 (Figure 3) [4]. Excavation was confined to undisturbed areas and undertaken in 2–3 cm excavation units to a maximum depth of 220 cm. Location features, including stratigraphic units and in situ finds, were mapped and recorded. Excavated material was wet screened and passed through 4 mm and 2 mm nested mesh sieves. 

### 2.2. Sampling 

**Figure 4:**
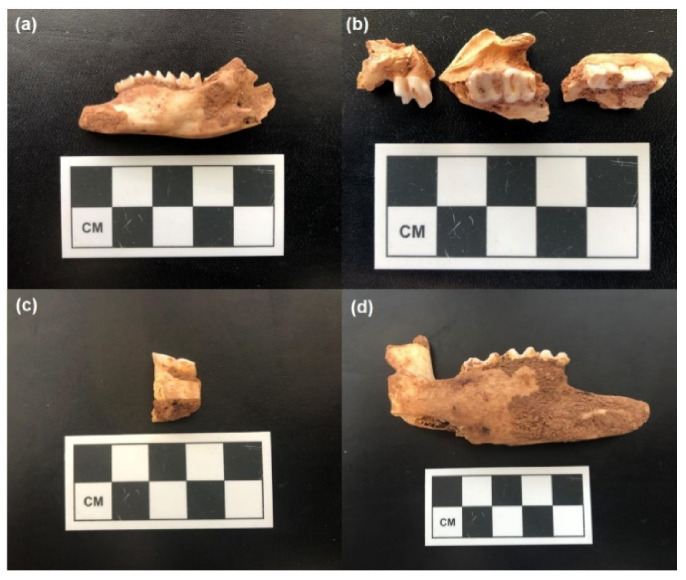
Examples of archaeological macropod teeth used for isotopic analysis from Boodie Cave: (**a**) right mandible fragment of *O. robustus* containing P3, M1, M2, M3 (from square G100, stratigraphic unit 3, excavation unit 510); (**b**) left maxilla fragments of *O. robustus* containing P3, M1, M2, M3 and M4 (from square G101, stratigraphic unit 3, excavation unit 534); (**c**) left mandible fragment of *L. conspicillatus* containing M2 and M3 (from square G101, stratigraphic unit 3, excavation unit 534); (**d**) left mandible fragment of *L. conspicillatus* containing M2, M3, and M4 (from square F101, stratigraphic unit 3, excavation unit 409).

Archaeological (*n* = 56) and modern (*n* = 13) *L. conspicillatus* and *O. robustus* teeth were collected from the Boodie Cave archaeological site on Barrow Island Western Australia between 2013 and 2015 (Figure 4). 

Archaeological specimens were identified to taxa and element at the University of Queensland School of Social Science Zooarchaeology laboratory. A modern comparative collection of several (deceased) individuals collected from Barrow Island between 2013 and 2014 facilitated identifications. All specimens were weighed and measured prior to pre-analytical processing. The archaeological material is between ~7 and 50 ka BP [4,5]. 

### 2.3. Preanatyical Processing and Pre-Treatments 

Preanalytical processing was conducted at the University of Western Australian School of Social Science Archaeology laboratory in accordance with contemporary methods [13,22,23,24]. Tooth enamel from whole teeth was separated from dentine using a Micro Dremel drill fitted with diamond coated drill-bits and subsequently ground into a fine powder using an agate mortar and pestle. 

Powdered tooth enamel samples were treated overnight with 50 μL of 3% hydrogen peroxide per 1.0 mg of enamel to remove organic matter. This was followed by a fifteen-minute treatment with 0.1 M acetic acid (50 μL of per 1.0 mg of sample) to remove diagenetic and absorbed carbonate. After each treatment, samples were rinsed with demineralized water, centrifuged four times, and dried in a desiccator.

### 2.4. Isotopic Analysis 

Isotopic values are given in per mil (‰) difference in the ratio of heavier to lighter isotopes (R) compared to that of a standard and are expressed using the standard delta notion (δ) as follows: Rsample Rstandard –

(1) $$\delta =\left[\left(\frac{\mathrm{Rsample}}{\mathrm{Rstandard}}-1\right)\right]\times \;1000$$

Given that the carbonate phase of macropod tooth enamel is c. 5%, approximately 6 mg samples of treated enamel (0.3 mg of carbonate) were analyzed for δ^13^C and δ^18^O. Analyses were undertaken using an GasBench II coupled with Delta XL Mass Spectrometer (Thermo-Fisher Scientific, Bremen, Germany) at the West Australian Biochemistry Centre, School of Plant Biology, University of Western Australia [25]. The isotope results were standardized to the Vienna PeeDee Belemnite (VPDB) and given in per mil (‰). Three-points normalization was used in order to reduce raw values to the international scale [26] and normalization was done based on international standards provided by IAEA: LSVEC, NBS19 and NBS18. The external error of δ^13^C analyses is <0.10‰ and δ^18^O is <0.10‰ (1 sd = standard deviation). 

### 2.5. Statistical Analysis 

Statistical visualizations and analyses were conducted using R. Pairwise Wilcoxon rank sum tests. *p*-values corrected for multiple comparisons are presented. 

## 3. Results

### 3.1. Carbon Isotopes 

**Figure 5:**
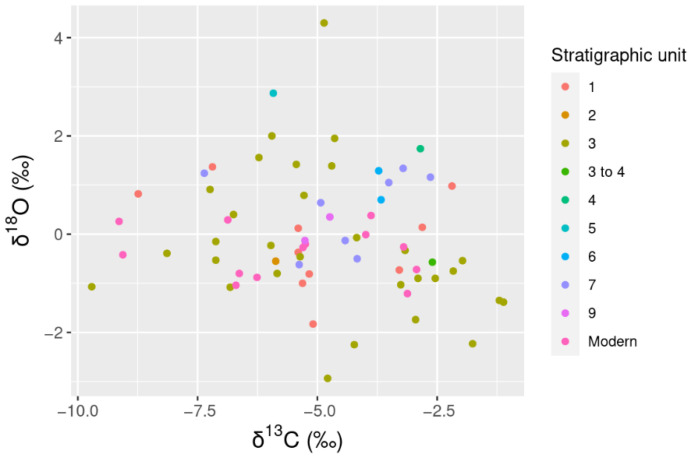
δ^18^O and δ^13^C for *L. conspicillatus* and *O. robustus.*

**Figure 6:**
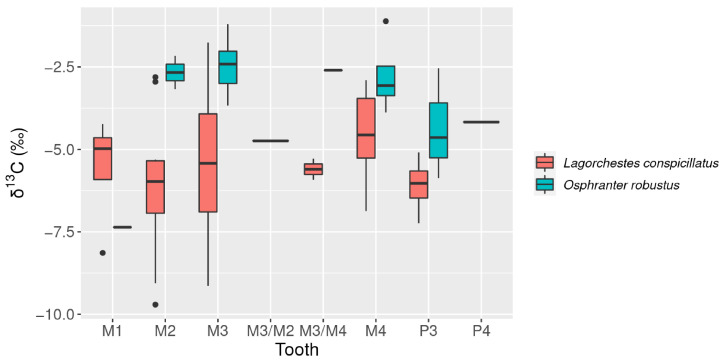
Box plot *L. conspicillatus* and *O. robustus* δ^13^C by tooth (M = molar, P = premolar).

Isotopic measurements were recorded for a total of 69 *L. conspicillatus* and *O. robustus* samples. All isotopic results are presented in Table S1 (Supplementary Material). Species mean isotopic results and standard deviations are summarized in Table 1. Overall δ^13^C values range from −9.71 ‰ to −1.11‰ (Figure 5). The adjusted *p*-value (0.00004) implies a statistically significant difference between the δ^13^C for *L. conspicillatus* (mean = −5.47‰, sd = 1.80) and *O. robustus* (mean =−3.42‰, sd =1.57). In general, late formed molars (M3 and M4) recorded higher δ^13^C values than those formed prior to weaning (M1 and M2) (Figure 6). This aligns with a known weaning effect [16].

**Figure 7:**
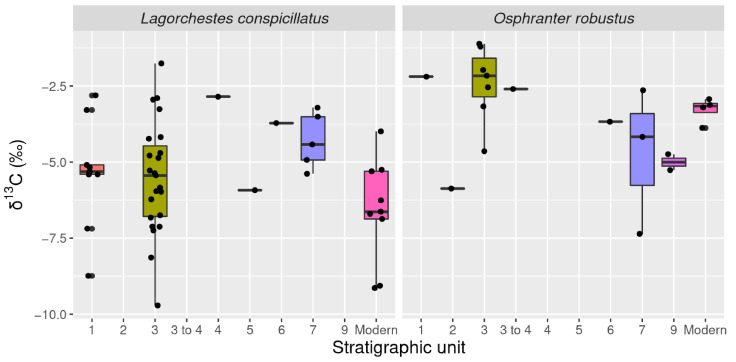
Box plot *L. conspicillatus* and *O. robustus* δ^13^C by stratigraphic unit.

Stratigraphic units, and corresponding chronological, paleoenvironmental and archeological contexts for Boodie Cave are documented in recent syntheses and summarized in Table 2 [4,5,27]. Comparison did not identify a statistically discernible separation between the archaeological and modern teeth in this study (Figure 7). Equally, no significant inter-strata differences were identified. This is true even when a −1.2‰ correction is added to the archaeological specimens to account for depletion in modern δ^13^C resulting from burning of fossil fuels [28,29]. While no statistically significant differences were detected between stratigraphic units, δ^13^C associated with the islandization phase trended more negative than signals recorded in layers linked to regression of the shoreline with falling sea levels.

### 3.2. Oxygen Isotopes 

**Figure 8:**
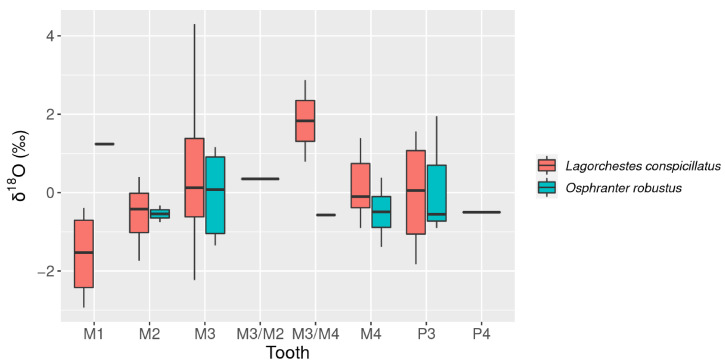
Box plot *L. conspicillatus* and *O. robustus* δ^18^O by tooth (M = molar, P = premolar).

Measured δ^18^O is statistically indistinguishable between *L. conspicillatus* (mean = 0.01‰, sd = 1.33) and *O. robustus* (mean = −0.12‰, sd = 0.93) (Table 1). Overall δ^18^O values range from −2.94‰ to 4.30‰ (Figure 5). This range overlaps the known modern δ^18^O values for *Macropus* spp. and *Osphranter* spp. in the wider Carnarvon bioregion (−2.07 to 5.62‰) [17]. Inter-tooth comparison did not identify any notable trends (Figure 8). 

**Figure 9:**
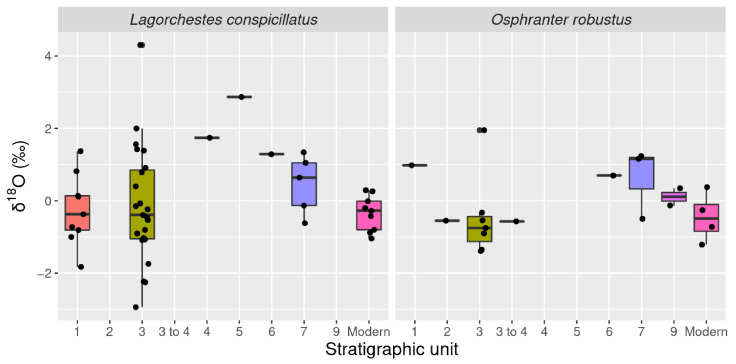
Box plot *L. conspicillatus* and *O. robustus* δ^18^O by stratigraphic unit.

**Figure 10:**
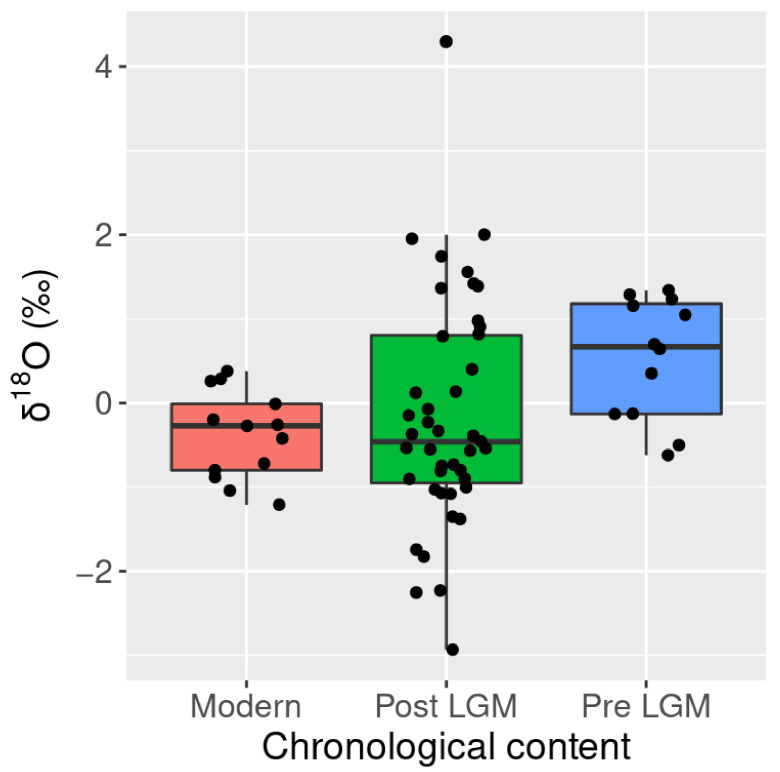
Box plot *L. conspicillatus* and *O. robustus* δ^18^O by climatic period.

Inter-stratigraphic analysis identified several significant differences in δ^18^O. Stratigraphic units six (mean = 0.10‰, sd = 0.42) and seven (mean = 0.52‰, sd = 0.82) are both statistically distinct from the modern specimens (mean = −0.375 ‰, sd = 0.52) (adjusted *p*-values = 0.86; 0.88) (Figure 9). More broadly, when stratigraphic units were analyzed by chronological context, Late Pleistocene samples pre-dating the Last Glacial Maximum (LGM) (mean = 0.53‰, sd = 0.72) were significantly different when compared to the post LGM Holocene group (mean = −0.17‰, sd = 1.37) (adjusted *p*-value = 0.08) (Figure 10). Similarly, a statistically notable shift was recorded between modern (mean = −0.38‰, sd = 0.52) and the pre-LGM samples (mean = 0.53‰, sd = 0.72) (adjusted *p*-value = 0.01). 

## 4. Discussion

### 4.1. Feeding Ecology and Paleo-Vegetation 

Based on the measured enamel δ^13^C and the known fractionation factor of approximately 12‰ [16], dietary δ^13^C for macropods in the study ranged from −21.71‰ to −13.11‰. This implies that the sampled taxa subsisted on a mixed C3 and C4 diet (Table 3) [30,31]. Notably, a clear separation is evident between *L. conspicillatus* and *O. robustus*. Although this difference may be influenced by the smaller number of *O. robustus* samples, it more likely reflects a dietary divergence. While both subsist on a predominantly C4 diet, results suggest that *L. conspicillatus* incorporates more C3 into its diet. 

Given that 70% of Barrow Island, including the area surrounding Boodie Cave, is characterized by limestone uplands dominated by C4 *Triodia wiseana* [3], this is interpreted to represent divergent behavioral and feeding strategies rather than distinct habitats. In particular, larger kangaroos species, including *O. robustus*, are widely regarded as preferential grazers that intermittently revert to browsing in arid habitats [32,33,34,35].

Conversely, although less is known about the feeding ecology of *L. conspicillatus*, it is understood that it is a selective feeder prone to browse on a mixture of monocotyledonous and dicotyledonous species including the tips of *Triodia* spp., shrub foliage and dicot herbs [36,37,38,39]. 

While no statistically significant differences were detected between archaeological strata, a number of noteworthy features and trends were identified in the data. In particular, *L. conspicillatus* δ^13^C associated with the island and islandization phases trended more negative than signals recorded in layers linked to regression of the shoreline, while *O. robustus* signals were more positive in the transgression phase. That is, there is a reversal in the direction of change between species. Given that the island and islandization phases are associated with a climatic period broadly defined by ameliorated conditions (including warmer temperatures, increased precipitation, and improved biological productivity) [27,40,41], it is posited that macropods had access to a wider range of lush vegetation and therefore their opposing tendencies toward graze and browse based diets were magnified due to these environmental shifts. 

Even when inter-species difference is taken into account, it is clear that δ^13^C recorded for modern teeth and enamel from more recent archaeological strata (SU1-3) are highly variable compared to earlier pre-LGM layers. Although the noisier signal can be partially attributed to sample size, it is probable that it reflects increased diversity in diet and potentially also in vegetation structure. This is likely the result of heightened variation in biological productivity in the arid zone during the Holocene and increases in sub annual fluctuations in rainfall at the onset of Marine Isotope Stage (MSI) 1 [27,40,42]. Given that C3 and C4 plants have different sensitivity to water availability, diversity of diet also likely contributed to variability in Holocene δ^18^O. 

### 4.2. Paleohumidity 

The lack of discernible statistical difference between δ^18^O for *L. conspicillatus* and *O. robustus* supports the expectation that the taxa are utilizing comparable water sources to meet their hydration requirements. Given the lack of free water sources on Barrow Island [43], it is hypothesized that the non-obligate drinkers are primarily obtaining hydration from dietary plant matter [39,44]. In addition, the lack of discernible difference between teeth aligns with existing data implying the absence of a weaning effect for δ^18^O and allows for species and molars to be analyzed in bulk [17].

δ^18^O exhibited statistically significant separation between modern samples and the strata associated with the pre-LGM regressing shoreline (SU 6 and SU 7) between 36.6 and 46.2 ka BP. On the basis that the vast majority of physiological and environmental factors that may influence the signal are negated by the environment, this difference is interpreted to reflect changes in relative humidity. 

Broadly, the higher δ^18^O associated with late Pleistocene samples implies drier conditions and while contradicting some existing continental and regional models that suggest the late MIS 3 between 40 and 30 ka was predominantly characterized by a humid phase and wetter conditions than the present [27,45], a more recent synthesis by De Deckker et al. 2020 [46] is consistent with significantly increasing aridity leading into the LGM. Conditions were highly variable across the continent and the onset of increasingly arid and cool conditions has been noted in the northern Australia from 35 ka [41]. This variability attests to the relevance of seeking proximal archaeological sources for interpreting paleoenvironments. 

In addition, Holocene (post-LGM) δ^18^O was significantly more negative than the late Pleistocene samples. Given that many Holocene samples are derived from stratigraphic unit three and date to between 6.8 and 7.2 ka BP, it is probable that this result aligns with the earliest Holocene low δ^18^O values identified in the regional Cape Range stalagmite record, coinciding with southern Australia mega-lake highstands [1]. Significantly, the archaeology in this period is exceptionally rich indicating intensive site use associated with a humid phase [4]

Importantly, results do not appear to reflect the interaction between mean temperature and relative humidity established by Murphy et al. 2007 [17] but are more strongly linked to known changes in levels of humidity [27,41] 

## 5. Conclusions

Paleoenvironmental signatures in archaeological and modern teeth from the Boodie Cave archaeological site on Barrow Island track shifting climatic conditions and, to a lesser extent, evolving habitats. Shifting δ^13^C potentially implies a slow but progressive increase in vegetation diversity associated with broader climatic amelioration. 

Significant inter-strata variation in δ^18^O between modern, Holocene, and late Pleistocene samples broadly aligns with continental and regional models but also contributes to a locate climate record. High δ^18^O values between 36.6 and 46.2 ka BP demonstrates the local onset of pre-LGM aridity and an early to mid-Holocene low in δ^18^O aligns with an increasingly humid period reflected in regional speleothem records, consistent with existing climate models [1,46]. 

Fundamentally, this study demonstrates for the first time that isotopic analysis of macropod tooth enamel can be used to track paleoenvironmental shifts to inter-strata resolution in Australian archaeological contexts. 

## Figures and Tables

**Figure 2:**
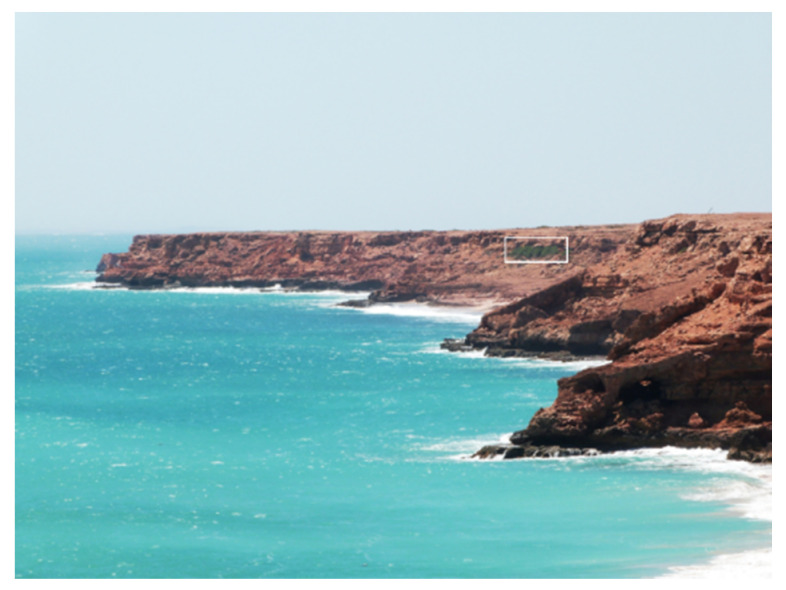
Photograph of Boodie Cave highlighting southwest entrance (Photograph by Peter Veth).

**Table 1:** Mean stable carbon and oxygen data for modern and archaeological *L. conspicillatus* and ^2^*O. robustus* tooth enamel carbonates.

Species	Type	n	δ^13^C ^1^	sd	δ^18^O ^1^	sd
*L. conspicillatus*	Archaeological	40	−5.22	1.74	−0.07	1.45
	Modern	9	−6.58	1.70	−0.34	0.49
*O. robustus*	Archaeological	16	−3.46	1.76	−0.04	0.99
	Modern	4	−3.28	0.41	−0.45	0.68

^1^ All δ^13^C and δ^18^O values given in per mil [‰, VPDB]. The external error of δ^13^C analyses is < 0.10‰ and the external error of δ^18^O is < 0.10‰ (1 sd). 2 sd = standard deviation.

**Table 2:** Stratigraphic unit and corresponding chronological and paleoenvironmental context.

Stratigraphic Unit	Chronology (ka BP) ^1^	Local Paleoenvironmental Context ^1^	Regional Climate Context ^2^
1	1.7–2.5	Fully marine (island)	MIS1, Holocene variability
2	2.5−6.8	Fully marine (island)	
3	6.8–7.2	Islandization	
4	7.2–7.4	Islandization	
5	7.4–22.4	Transgressing shoreline	Start of MIS1 at 14 ka, deglacial variability
		Discontinuity	MIS2, LGM aridity and complex glacial response
6	36.6–42.6	Regressing Shoreline	Late MIS3, humid phase, and initiation of cooler conditions
7	42.6–46.2	Regressing Shoreline	
		Discontinuity	MIS3
8	46.2–51.1	Transgressing Shoreline; cave opens	MIS3
9	~77	Continental–regressing shoreline; cave closed	MIS4

^1^ Based on existing archaeological and paleoenvironmental syntheses of Boodie Cave [4,5]; ^2^ Based on synthesis of existing paleoenvironmental data from Australian continent [27].

**Table 3:** δ^13^C_enamel_, δ^13^C_diet and_ %C3 diet for archaeological and modern taxa.

Species	Type	δ^13^C_enamel_	δ^13^C_diet_^1^	%C3 Diet ^2^
*L. conspicillatus*	Archaeological	−5.22	−17.22	23.00%
	Modern	−6.58	−18.58	32.71%
*O. robustus*	Archaeological	−3.46	−15.46	10.43%
	Modern	−3.28	−15.28	9.14%

^1^ δ^13^C_diet_ is calculated by subtracting the known diet-enamel enrichment factor of 12‰ from δ^13^C_enamel._; ^2^ %C3 diet in calculated using the following mixing equation: f_1_ = (δ_SAMPLE-_δ_SOURCE2_)/(δs_OURCE1-_δ_SOURCE2_) × 100 where source 1 is the average δ^13^C for C3 vegetation (−28‰) and source 2 is the average δ^13^C for C4 (−14‰) [30,31].

## Data Availability

Not applicable.

## Supplementary Materials

The following is available online: Table S1: Isotopic results for *L. conspicillatus* and *O. robustus*.

## Sample Availability

Samples of the compounds are not available from the authors.
