# Supplementary material for: Isotopic Indications of Late Pleistocene and Holocene Paleoenvironmental Changes at Boodie Cave Archaeological Site, Barrow Island, Western Australia
Source: Molecules. 2021 Apr 28;26(9):2582. doi: 10.3390/molecules26092582 (PMC8124622; doi:10.3390/molecules26092582)
Supplement: Supplementary file 1 [file molecules-26-02582-s001.zip › molecules-1171790-supplementary.pdf]

Table S1. Isotopic results for *L. conspicillatus* and *O. robustus*.

Species	Tooth	Stratigraphic Unit	δ¹³C [‰, VPDB]	δ¹⁸O [‰, VPDB]
Lagorchestes conspicillatus	M4	1	−3.29	−0.73
Lagorchestes conspicillatus	M2	1	−2.81	0.14
Lagorchestes conspicillatus	M3	1	−7.19	1.37
Lagorchestes conspicillatus	M2	1	−5.40	0.12
Lagorchestes conspicillatus	M3	1	−8.74	0.82
Lagorchestes conspicillatus	P3	1	−5.09	−1.83
Lagorchestes conspicillatus	M1	1	−5.17	−0.81
Lagorchestes conspicillatus	M2	1	−5.31	−1.00
Lagorchestes conspicillatus	M3	1	−5.40	−0.37
Lagorchestes conspicillatus	M3	3	−3.26	−1.03
Lagorchestes conspicillatus	P3	3	−5.84	−0.80
Lagorchestes conspicillatus	M1	3	−4.23	−2.25
Lagorchestes conspicillatus	M2	3	−2.95	−1.74
Lagorchestes conspicillatus	M3	3	−7.12	−0.53
Lagorchestes conspicillatus	M4	3	−2.90	−0.90
Lagorchestes conspicillatus	M1	3	−8.14	−0.39
Lagorchestes conspicillatus	M2	3	−7.12	−0.15
Lagorchestes conspicillatus	M3	3	−5.95	2.00
Lagorchestes conspicillatus	M3	3	−1.76	−2.23
Lagorchestes conspicillatus	M4	3	−4.18	−0.07
Lagorchestes conspicillatus	M3	3	−5.36	−0.46
Lagorchestes conspicillatus	M2	3	−9.71	−1.07
Lagorchestes conspicillatus	M3	3	−6.82	−1.08
Lagorchestes conspicillatus	P3	3	−6.22	1.56
Lagorchestes conspicillatus	L2	3	−5.97	−0.23
Lagorchestes conspicillatus	M3	3	−4.86	4.30
Lagorchestes conspicillatus	M4	3	−4.70	1.39
Lagorchestes conspicillatus	P3	3	−7.24	0.91
Lagorchestes conspicillatus	M1	3	−4.79	−2.94
Lagorchestes conspicillatus	M2	3	−6.75	0.40
Lagorchestes conspicillatus	M3	3	−5.44	1.42
Lagorchestes conspicillatus	M3/M4	3	−5.28	0.79
Lagorchestes conspicillatus	M3	4	−2.85	1.74
Lagorchestes conspicillatus	M3/M4	5	−5.92	2.87
Lagorchestes conspicillatus	M3	6	−3.72	1.29
Lagorchestes conspicillatus	M4	7	−3.21	1.34
Lagorchestes conspicillatus	M4	7	−4.42	−0.13
Lagorchestes conspicillatus	M4	7	−3.51	1.05
Lagorchestes conspicillatus	M2	7	−5.38	−0.62
Lagorchestes conspicillatus	M4	7	−4.93	0.64
Lagorchestes conspicillatus	M4	Modern	−6.87	0.29
Lagorchestes conspicillatus	M3	Modern	−9.14	0.26
Lagorchestes conspicillatus	M2	Modern	−9.06	−0.42
Lagorchestes conspicillatus	M3	Modern	−3.99	−0.01
Lagorchestes conspicillatus	M4	Modern	−5.30	−0.27
Lagorchestes conspicillatus	M4	Modern	−6.63	−0.80
Lagorchestes conspicillatus	M4	Modern	−5.25	−0.20
Lagorchestes conspicillatus	M3	Modern	−6.26	−0.88

Lagorchestes conspicillatus	M2	Modern	−6.70	−1.04
Osphranter robustus	M3	1	−2.19	0.98
Osphranter robustus	P3	2	−5.87	−0.55
Osphranter robustus	P3	3	−4.64	1.95
Osphranter robustus	M2	3	−3.17	−0.33
Osphranter robustus	M3	3	−1.97	−0.54
Osphranter robustus	P3	3	−2.54	−0.90
Osphranter robustus	M2	3	−2.16376	−0.75073
Osphranter robustus	M3	3	−1.2023	−1.34868
Osphranter robustus	M4	3	−1.11398	−1.38363
Osphranter robustus	M3/4	3 to 4	−2.60	−0.57
Osphranter robustus	M3	6	−3.67	0.70
Osphranter robustus	P4	7	−4.17	−0.50
Osphranter robustus	M1	7	−7.36	1.24
Osphranter robustus	M3	7	−2.64	1.16
Osphranter robustus	M3/M2	9	−4.74	0.35
Osphranter robustus	?	9	−5.26	−0.13
Osphranter robustus	M4	Modern	−3.20	−0.26
Osphranter robustus	M3	Modern	−3.12	−1.21
Osphranter robustus	M4	Modern	−2.93	−0.72
Osphranter robustus	M4	Modern	−3.88	0.38
